# Bis[(2,2-dimethyl­propano­yloxy)meth­yl] {[2-(6-amino-9*H*-purin-9-yl)eth­oxy]meth­yl}phospho­nate–succinic acid (2/1)

**DOI:** 10.1107/S1600536812006873

**Published:** 2012-02-24

**Authors:** Sungyup Jung, Jeong-Myeong Ha, Il Won Kim

**Affiliations:** a Department of Chemical Engineering, Soongsil University, 369 Sangdo-ro, Dongjak-gu, Seoul 156-743, Republic of Korea; b Korea Institute of Science and Technology, Hwarangno 14-gil 5, Seongbuk-gu, Seoul 136-791, Republic of Korea

## Abstract

The title compound, C_20_H_32_N_5_O_8_P·0.5C_4_H_6_O_4_, is composed of two 9-{2-[bis­(pivaloyloxymeth­oxy)phosphinylmeth­oxy]eth­yl}adenine, commonly known as adefovir dipivoxil (AD), mol­ecules linked to the carb­oxy­lic acid groups of succinic acid (SA). The asymmetric unit contains one mol­ecule of AD and half a mol­ecule of SA, which sits on an inversion center. Both adenine units in the two AD mol­ecules make AD–SA N—H⋯O and SA–AD O—H⋯N hydrogen bonds to SA. In addition, the inter­molecular AD–AD N—H⋯O—P hydrogen bond serves to stabilize the cocrystal. There is also a π–π stacking inter­action [inter­planar spacing 3.34 (19) Å] between adjacent inversion-related adenine groups.

## Related literature

For the synthesis and process optimization of 9-{2-[bis(pivaloyloxymeth­oxy)phosphinylmeth­oxy]eth­yl}adenine, see: Starrett *et al.* (1992 ▶); Yu *et al.* (1999 ▶). For the biological and pharmacological relevance of 9-{2-[bis­(pivaloyloxymeth­oxy)phosphinylmeth­oxy]eth­yl}adenine, see: Qaqish *et al.* (2003 ▶); Julander *et al.* (2002 ▶). For the structure of a hydrate of the title compound, see: Chang *et al.* (2007 ▶).
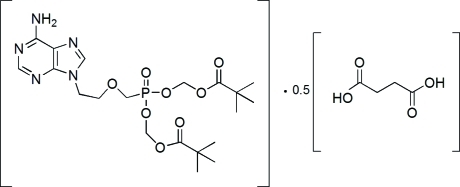


## Experimental

### 

#### Crystal data

C_20_H_32_N_5_O_8_P·0.5C_4_H_6_O_4_
*M* *_r_* = 560.52
Triclinic, 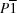

*a* = 7.7122 (12) Å
*b* = 10.1577 (15) Å
*c* = 19.185 (3) Å
α = 80.409 (8)°
β = 79.718 (9)°
γ = 80.407 (8)°
*V* = 1443.5 (4) Å^3^
*Z* = 2
Mo *K*α radiation
μ = 0.15 mm^−1^
*T* = 296 K
0.11 × 0.10 × 0.08 mm

#### Data collection

Bruker SMART CCD diffractometer
Absorption correction: multi-scan (*SADABS*; Bruker, 2002 ▶) *T*_min_ = 0.982, *T*_max_ = 0.987
49737 measured reflections
7222 independent reflections
4593 reflections with *I* > 2σ(*I*)
*R*_int_ = 0.053

#### Refinement

*R*[*F* ^2^ > 2σ(*F* ^2^)] = 0.046
*wR*(*F* ^2^) = 0.143
*S* = 1.01
7222 reflections
417 parameters
H atoms treated by a mixture of independent and constrained refinement
Δρ_max_ = 0.38 e Å^−3^
Δρ_min_ = −0.29 e Å^−3^

### 

Data collection: *SMART* (Bruker, 2002 ▶); cell refinement: *SAINT* (Bruker, 2002 ▶); data reduction: *SAINT*; program(s) used to solve structure: *SHELXS97* (Sheldrick, 2008 ▶); program(s) used to refine structure: *SHELXL97* (Sheldrick, 2008 ▶); molecular graphics: *ORTEP-3 for Windows* (Farrugia, 1997 ▶); software used to prepare material for publication: *WinGX* (Farrugia, 1999 ▶).

## Supplementary Material

Crystal structure: contains datablock(s) I, global. DOI: 10.1107/S1600536812006873/pk2386sup1.cif

Structure factors: contains datablock(s) I. DOI: 10.1107/S1600536812006873/pk2386Isup2.hkl

Supplementary material file. DOI: 10.1107/S1600536812006873/pk2386Isup3.cml

Additional supplementary materials:  crystallographic information; 3D view; checkCIF report

## Figures and Tables

**Table 1:** Hydrogen-bond geometry (Å, °)

*D*—H⋯*A*	*D*—H	H⋯*A*	*D*⋯*A*	*D*—H⋯*A*
N1—H1*C*⋯O1^i^	0.812 (19)	2.14 (2)	2.941 (2)	170.16 (18)
N1—H1*B*⋯O9	0.79 (2)	2.05 (2)	2.842 (2)	175 (2)
O10—H10⋯N4	0.84 (2)	1.91 (2)	2.734 (2)	166 (2)

Symmetry code: (i) 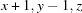.

**Fig. 1.**
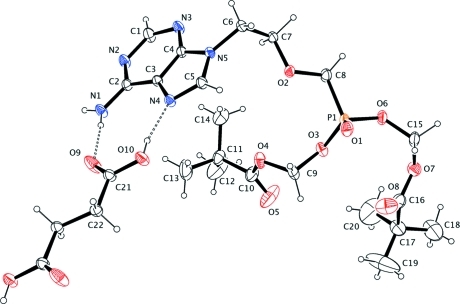
The molecular structure of the title compound, showing 20% probability displacement ellipsoids. H atoms are shown as small spheres of arbitrary radius. Intermolecular interactions are shown as dashed lines. [Symmetry code: (i) -x + 2, -y - 1, -z].

**Fig. 2.**
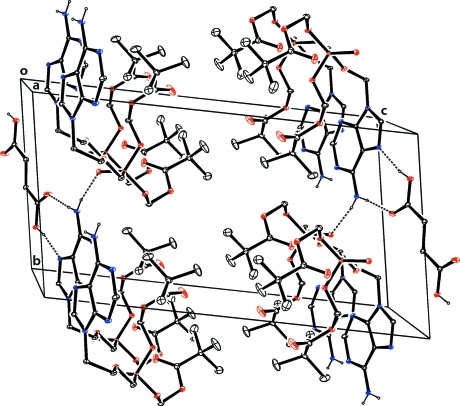
Crystal packing diagram for the title compound. For clarity, H atoms are shown only for those involved in hydrogen bonding (dashed lines).

## supplementary crystallographic information

### Comment

9-{2-[Bis(pivaloyloxymethoxy)phosphinylmethoxy]ethyl}adenine, also known as adefovir dipivoxil (AD), is a broad-spectrum antiviral from the class of acyclic nucleoside phosphonates. It is an orally bioavailable prodrug of 9-[2-(phosphonylmethoxy)ethyl] adenine, which acts as a chain terminator nucleotide analogue and is effective against the human immunodeficiency virus, herpes viruses, Epstein–Barr virus, retroviruses, cytomegalovirus, and other DNA viruses (Yu *et al.*, 1999; Julander *et al.*, 2002; Qaqish *et al.*, 2003). In the present study, we report a new cocrystal of AD with succinic acid to later study the physical characteristics, such as thermal stability and *in vitro* release behavior.

### Experimental

The title compound was formed during cocrystallization in a 2:1 molar ratio of 9-{2-[bis(pivaloyloxymethoxy)phosphinylmethoxy]ethyl}adenine, commonly known as adefovir dipivoxil, (0.4 mmol, AMoRe Pacific Co., purity > 99%) and succinic acid (0.2 mmol, Sigma–Aldrich, purity > 99%). The two components were dissolved in ethanol (3 ml, Samchun Chemical, Korea, HPLC grade) and heated at 45–50°C for 1 h. The prepared solution was stored in a 25°C incubator, and the crystals were started to be visible after about 1 d. After 2 more weeks, the crystals were filtered, washed with deionized water (Resistivity > 18.2 MΩ-cm; Direct-Q, Millipore), and dried for 24 h in a 40°C vacuum oven.

### Refinement

All H atoms were located in a difference map. Methyl hydrogens on the *tert*-butyl carbons were positioned with idealized geometry using a riding model with C—H = 0.96 Å and U_iso_ = 1.5U_eq_ (C_Me_). All other hydrogens were freely refined.

### Crystal data

**Table:**

C_20_H_32_N_5_O_8_P·0.5C_4_H_6_O_4_	*Z* = 2
*M**_r_* = 560.52	*F*(000) = 594
Triclinic, *P*1	*D*_x_ = 1.290 Mg m^−^^3^
Hall symbol: -P 1	Melting point: 410 K
*a* = 7.7122 (12) Å	Mo *K*α radiation, λ = 0.71073 Å
*b* = 10.1577 (15) Å	Cell parameters from 9846 reflections
*c* = 19.185 (3) Å	θ = 2.2–25.7°
α = 80.409 (8)°	µ = 0.15 mm^−^^1^
β = 79.718 (9)°	*T* = 296 K
γ = 80.407 (8)°	Block, colourless
*V* = 1443.5 (4) Å^3^	0.11 × 0.10 × 0.08 mm

### Data collection

**Table:**

Bruker SMART CCD diffractometer	7222 independent reflections
Radiation source: fine-focus sealed tube	4593 reflections with *I* > 2σ(*I*)
Graphite monochromator	*R*_int_ = 0.053
φ and ω scans	θ_max_ = 28.4°, θ_min_ = 1.1°
Absorption correction: multi-scan (*SADABS*; Bruker, 2002)	*h* = −10→10
*T*_min_ = 0.982, *T*_max_ = 0.987	*k* = −13→13
49737 measured reflections	*l* = −25→25

### Refinement

**Table:**

Refinement on *F*^2^	Primary atom site location: structure-invariant direct methods
Least-squares matrix: full	Secondary atom site location: difference Fourier map
*R*[*F*^2^ > 2σ(*F*^2^)] = 0.046	Hydrogen site location: inferred from neighbouring sites
*wR*(*F*^2^) = 0.143	H atoms treated by a mixture of independent and constrained refinement
*S* = 1.01	*w* = 1/[σ^2^(*F*_o_^2^) + (0.0783*P*)^2^] where *P* = (*F*_o_^2^ + 2*F*_c_^2^)/3
7222 reflections	(Δ/σ)_max_ < 0.001
417 parameters	Δρ_max_ = 0.38 e Å^−^^3^
0 restraints	Δρ_min_ = −0.29 e Å^−^^3^

### Special details

**Table:**

Geometry. All e.s.d.'s (except the e.s.d. in the dihedral angle between two l.s. planes) are estimated using the full covariance matrix. The cell e.s.d.'s are taken into account individually in the estimation of e.s.d.'s in distances, angles and torsion angles; correlations between e.s.d.'s in cell parameters are only used when they are defined by crystal symmetry. An approximate (isotropic) treatment of cell e.s.d.'s is used for estimating e.s.d.'s involving l.s. planes.
Refinement. Refinement of *F*^2^ against ALL reflections. The weighted *R*-factor *wR* and goodness of fit *S* are based on *F*^2^, conventional *R*-factors *R* are based on *F*, with *F* set to zero for negative *F*^2^. The threshold expression of *F*^2^ > σ(*F*^2^) is used only for calculating *R*-factors(gt) *etc*. and is not relevant to the choice of reflections for refinement. *R*-factors based on *F*^2^ are statistically about twice as large as those based on *F*, and *R*-factors based on ALL data will be even larger.

### Fractional atomic coordinates and isotropic or equivalent isotropic displacement parameters (Å^2^)

**Table:**

	*x*	*y*	*z*	*U*_iso_*/*U*_eq_	
P1	0.85354 (6)	0.35488 (4)	0.23364 (3)	0.04127 (14)	
O2	1.16908 (15)	0.27477 (11)	0.16611 (7)	0.0465 (3)	
H22A	0.869 (3)	−0.400 (2)	0.0252 (13)	0.083 (8)*	
O1	0.75924 (17)	0.37739 (12)	0.17242 (7)	0.0520 (3)	
H7A	1.410 (3)	0.2107 (19)	0.1924 (10)	0.052 (5)*	
N4	1.30471 (18)	−0.10694 (13)	0.07213 (8)	0.0408 (3)	
H6A	1.364 (3)	0.2683 (19)	0.0495 (10)	0.050 (5)*	
N5	1.40907 (18)	0.07897 (12)	0.08442 (7)	0.0376 (3)	
H8A	1.137 (3)	0.353 (2)	0.2554 (13)	0.077 (7)*	
O10	1.08166 (19)	−0.23084 (12)	0.01701 (8)	0.0561 (4)	
H1C	1.590 (3)	−0.454 (2)	0.1269 (10)	0.047 (5)*	
N1	1.5210 (2)	−0.38651 (15)	0.11658 (10)	0.0497 (4)	
H5	1.177 (3)	0.0849 (19)	0.0460 (10)	0.054 (5)*	
O6	0.79832 (17)	0.45855 (12)	0.28933 (7)	0.0545 (3)	
H7B	1.377 (3)	0.371 (2)	0.1459 (10)	0.057 (5)*	
O3	0.83198 (18)	0.21639 (11)	0.28310 (7)	0.0526 (3)	
H6B	1.549 (3)	0.2239 (18)	0.0706 (10)	0.054 (6)*	
C3	1.4627 (2)	−0.14293 (15)	0.09965 (9)	0.0350 (4)	
H1A	1.870 (3)	−0.1491 (19)	0.1681 (11)	0.059 (6)*	
N2	1.7135 (2)	−0.26489 (14)	0.14725 (9)	0.0508 (4)	
H15B	0.551 (3)	0.523 (2)	0.2714 (12)	0.071 (7)*	
N3	1.6779 (2)	−0.02193 (14)	0.13207 (9)	0.0513 (4)	
H15A	0.633 (3)	0.619 (2)	0.3206 (12)	0.078 (7)*	
C4	1.5280 (2)	−0.02841 (15)	0.10754 (9)	0.0372 (4)	
H22B	0.974 (3)	−0.383 (2)	−0.0530 (12)	0.065 (6)*	
O4	0.9500 (2)	0.00392 (13)	0.25970 (8)	0.0628 (4)	
H8B	1.104 (3)	0.456 (2)	0.1946 (12)	0.073 (7)*	
O7	0.5407 (2)	0.45750 (14)	0.37225 (8)	0.0645 (4)	
C5	1.2792 (2)	0.02556 (16)	0.06432 (10)	0.0415 (4)	
C2	1.5646 (2)	−0.26729 (15)	0.12075 (9)	0.0383 (4)	
H9B	0.689 (4)	0.071 (3)	0.2924 (16)	0.112 (10)*	
C6	1.4277 (3)	0.22069 (16)	0.08249 (11)	0.0438 (4)	
H1B	1.438 (3)	−0.3928 (19)	0.0988 (10)	0.044 (6)*	
C7	1.3572 (2)	0.27262 (18)	0.15218 (11)	0.0450 (4)	
H9A	0.792 (3)	0.126 (2)	0.2055 (14)	0.088 (8)*	
C22	0.9785 (3)	−0.42444 (17)	−0.00200 (12)	0.0436 (4)	
H10	1.161 (3)	−0.206 (2)	0.0348 (13)	0.081 (8)*	
C21	1.1118 (2)	−0.36274 (17)	0.02458 (10)	0.0445 (4)	
C8	1.0864 (3)	0.3663 (2)	0.21406 (13)	0.0505 (5)	
C1	1.7603 (3)	−0.14515 (19)	0.15018 (13)	0.0577 (5)	
O9	1.2352 (2)	−0.42677 (14)	0.05093 (11)	0.0873 (6)	
O5	0.8495 (3)	−0.0752 (3)	0.37084 (13)	0.1501 (12)	
C9	0.7978 (3)	0.10227 (19)	0.25713 (16)	0.0618 (6)	
C11	1.1398 (3)	−0.1700 (2)	0.31830 (12)	0.0678 (6)	
C15	0.6237 (3)	0.5252 (2)	0.30718 (14)	0.0625 (6)	
O8	0.4230 (3)	0.3366 (2)	0.31153 (9)	0.0959 (6)	
C16	0.4436 (3)	0.3614 (2)	0.36752 (12)	0.0608 (5)	
C10	0.9642 (3)	−0.0782 (2)	0.32119 (13)	0.0696 (6)	
C18	0.2912 (6)	0.3830 (4)	0.4914 (2)	0.1566 (19)	
H18A	0.1843	0.4337	0.4764	0.235*	
H18B	0.3726	0.4436	0.4936	0.235*	
H18C	0.2627	0.3324	0.5379	0.235*	
C14	1.2937 (4)	−0.0882 (3)	0.28699 (18)	0.1026 (10)	
H14A	1.2850	−0.0523	0.2380	0.154*	
H14B	1.4052	−0.1458	0.2894	0.154*	
H14C	1.2870	−0.0155	0.3140	0.154*	
C12	1.1638 (5)	−0.2356 (4)	0.39287 (18)	0.1525 (18)	
H12A	1.1480	−0.1675	0.4235	0.229*	
H12B	1.2813	−0.2852	0.3922	0.229*	
H12C	1.0774	−0.2960	0.4105	0.229*	
C17	0.3749 (4)	0.2886 (3)	0.43929 (13)	0.0794 (7)	
C20	0.5398 (7)	0.2122 (6)	0.4705 (3)	0.213 (3)	
H20A	0.5030	0.1645	0.5170	0.320*	
H20B	0.6162	0.2752	0.4746	0.320*	
H20C	0.6030	0.1491	0.4395	0.320*	
C19	0.2625 (9)	0.1901 (6)	0.4313 (2)	0.227 (3)	
H19A	0.2433	0.1300	0.4754	0.341*	
H19B	0.3207	0.1392	0.3937	0.341*	
H19C	0.1501	0.2367	0.4198	0.341*	
C13	1.1420 (4)	−0.2716 (3)	0.2684 (2)	0.1238 (13)	
H13A	1.2543	−0.3294	0.2651	0.186*	
H13B	1.1254	−0.2249	0.2217	0.186*	
H13C	1.0477	−0.3249	0.2866	0.186*	

### Atomic displacement parameters (Å^2^)

**Table:**

	*U*^11^	*U*^22^	*U*^33^	*U*^12^	*U*^13^	*U*^23^
P1	0.0401 (3)	0.0328 (2)	0.0502 (3)	0.00576 (17)	−0.0116 (2)	−0.01014 (19)
O2	0.0360 (7)	0.0422 (6)	0.0663 (8)	−0.0040 (5)	−0.0069 (6)	−0.0242 (6)
O1	0.0506 (8)	0.0463 (7)	0.0587 (8)	0.0086 (6)	−0.0214 (6)	−0.0078 (6)
N4	0.0401 (8)	0.0326 (7)	0.0523 (9)	−0.0019 (6)	−0.0169 (7)	−0.0061 (6)
N5	0.0391 (8)	0.0280 (6)	0.0449 (8)	−0.0019 (5)	−0.0073 (6)	−0.0046 (6)
O10	0.0544 (9)	0.0388 (7)	0.0831 (10)	−0.0083 (6)	−0.0301 (8)	−0.0090 (6)
N1	0.0500 (10)	0.0280 (7)	0.0766 (12)	−0.0011 (7)	−0.0322 (9)	−0.0034 (7)
O6	0.0524 (8)	0.0457 (7)	0.0661 (9)	0.0058 (6)	−0.0065 (7)	−0.0249 (6)
O3	0.0654 (9)	0.0379 (6)	0.0564 (8)	−0.0030 (6)	−0.0194 (7)	−0.0062 (6)
C3	0.0367 (9)	0.0315 (8)	0.0377 (9)	−0.0033 (6)	−0.0095 (7)	−0.0054 (6)
N2	0.0493 (9)	0.0369 (8)	0.0724 (11)	0.0008 (6)	−0.0288 (8)	−0.0117 (7)
N3	0.0483 (9)	0.0385 (8)	0.0751 (11)	−0.0039 (7)	−0.0245 (8)	−0.0172 (7)
C4	0.0382 (9)	0.0317 (8)	0.0426 (9)	−0.0018 (6)	−0.0082 (7)	−0.0087 (7)
O4	0.0758 (10)	0.0428 (7)	0.0635 (9)	0.0123 (6)	−0.0158 (8)	−0.0050 (6)
O7	0.0757 (10)	0.0612 (9)	0.0554 (9)	−0.0103 (7)	0.0005 (8)	−0.0159 (7)
C5	0.0416 (10)	0.0335 (8)	0.0498 (10)	−0.0018 (7)	−0.0131 (8)	−0.0042 (7)
C2	0.0400 (9)	0.0340 (8)	0.0420 (9)	−0.0027 (7)	−0.0111 (8)	−0.0058 (7)
C6	0.0421 (11)	0.0283 (8)	0.0588 (12)	−0.0039 (7)	−0.0047 (9)	−0.0038 (8)
C7	0.0353 (10)	0.0356 (9)	0.0676 (13)	−0.0042 (7)	−0.0089 (9)	−0.0167 (9)
C22	0.0392 (10)	0.0415 (9)	0.0544 (12)	−0.0054 (8)	−0.0170 (9)	−0.0090 (8)
C21	0.0420 (10)	0.0397 (9)	0.0553 (11)	−0.0078 (7)	−0.0155 (9)	−0.0066 (8)
C8	0.0455 (11)	0.0486 (11)	0.0624 (13)	−0.0021 (9)	−0.0105 (10)	−0.0245 (10)
C1	0.0504 (12)	0.0462 (10)	0.0869 (16)	0.0009 (9)	−0.0357 (11)	−0.0201 (10)
O9	0.0812 (11)	0.0438 (8)	0.1585 (17)	−0.0067 (7)	−0.0839 (12)	−0.0076 (9)
O5	0.1080 (17)	0.147 (2)	0.1177 (18)	0.0577 (15)	0.0455 (14)	0.0593 (15)
C9	0.0713 (15)	0.0328 (9)	0.0861 (17)	0.0026 (9)	−0.0317 (14)	−0.0108 (10)
C11	0.0641 (14)	0.0598 (13)	0.0673 (14)	0.0139 (11)	−0.0086 (11)	0.0018 (11)
C15	0.0613 (14)	0.0448 (11)	0.0703 (15)	0.0110 (10)	0.0058 (12)	−0.0123 (10)
O8	0.0926 (14)	0.1482 (18)	0.0600 (11)	−0.0470 (12)	−0.0158 (10)	−0.0182 (11)
C16	0.0598 (13)	0.0672 (13)	0.0557 (13)	−0.0030 (10)	−0.0095 (11)	−0.0147 (11)
C10	0.0685 (15)	0.0583 (13)	0.0648 (15)	0.0093 (11)	0.0023 (12)	0.0085 (11)
C18	0.227 (5)	0.119 (3)	0.102 (3)	−0.060 (3)	0.081 (3)	−0.034 (2)
C14	0.0750 (19)	0.108 (2)	0.119 (2)	−0.0001 (16)	−0.0038 (17)	−0.0247 (19)
C12	0.117 (3)	0.192 (4)	0.094 (2)	0.056 (3)	−0.010 (2)	0.051 (2)
C17	0.107 (2)	0.0736 (15)	0.0561 (14)	−0.0243 (15)	0.0029 (14)	−0.0100 (12)
C20	0.201 (5)	0.230 (5)	0.145 (4)	0.024 (4)	−0.028 (4)	0.104 (4)
C19	0.361 (8)	0.261 (6)	0.110 (3)	−0.248 (6)	0.027 (4)	−0.034 (3)
C13	0.095 (2)	0.0793 (19)	0.201 (4)	0.0341 (16)	−0.047 (2)	−0.053 (2)

### Geometric parameters (Å, º)

**Table:**

P1—O1	1.4562 (13)	C22—H22B	1.00 (2)
P1—O3	1.5760 (13)	C21—O9	1.200 (2)
P1—O6	1.5794 (13)	C8—H8A	0.93 (2)
P1—C8	1.787 (2)	C8—H8B	0.95 (2)
O2—C8	1.413 (2)	C1—H1A	0.96 (2)
O2—C7	1.424 (2)	O5—C10	1.179 (3)
N4—C5	1.314 (2)	C9—H9B	1.04 (3)
N4—C3	1.383 (2)	C9—H9A	0.99 (3)
N5—C5	1.354 (2)	C11—C12	1.504 (4)
N5—C4	1.3707 (19)	C11—C10	1.508 (3)
N5—C6	1.464 (2)	C11—C13	1.518 (4)
O10—C21	1.309 (2)	C11—C14	1.538 (4)
O10—H10	0.84 (3)	C15—H15B	0.96 (2)
N1—C2	1.328 (2)	C15—H15A	1.05 (2)
N1—H1C	0.81 (2)	O8—C16	1.186 (3)
N1—H1B	0.79 (2)	C16—C17	1.501 (3)
O6—C15	1.413 (2)	C18—C17	1.490 (4)
O3—C9	1.415 (2)	C18—H18A	0.9600
C3—C4	1.383 (2)	C18—H18B	0.9600
C3—C2	1.411 (2)	C18—H18C	0.9600
N2—C1	1.337 (2)	C14—H14A	0.9600
N2—C2	1.341 (2)	C14—H14B	0.9600
N3—C1	1.327 (2)	C14—H14C	0.9600
N3—C4	1.338 (2)	C12—H12A	0.9600
O4—C10	1.337 (2)	C12—H12B	0.9600
O4—C9	1.411 (2)	C12—H12C	0.9600
O7—C16	1.349 (3)	C17—C19	1.471 (5)
O7—C15	1.420 (3)	C17—C20	1.539 (5)
C5—H5	0.990 (19)	C20—H20A	0.9600
C6—C7	1.497 (3)	C20—H20B	0.9600
C6—H6A	0.90 (2)	C20—H20C	0.9600
C6—H6B	0.93 (2)	C19—H19A	0.9600
C7—H7A	1.012 (19)	C19—H19B	0.9600
C7—H7B	1.02 (2)	C19—H19C	0.9600
C22—C21	1.490 (2)	C13—H13A	0.9600
C22—C22^i^	1.507 (3)	C13—H13B	0.9600
C22—H22A	0.93 (3)	C13—H13C	0.9600
			
O1—P1—O3	113.85 (8)	O3—C9—H9B	104.5 (16)
O1—P1—O6	117.52 (7)	O4—C9—H9A	103.8 (15)
O3—P1—O6	101.92 (8)	O3—C9—H9A	108.4 (14)
O1—P1—C8	116.19 (10)	H9B—C9—H9A	120 (2)
O3—P1—C8	106.95 (9)	C12—C11—C10	109.1 (2)
O6—P1—C8	98.37 (8)	C12—C11—C13	112.7 (3)
C8—O2—C7	111.57 (13)	C10—C11—C13	108.6 (2)
C5—N4—C3	104.30 (13)	C12—C11—C14	109.0 (3)
C5—N5—C4	105.98 (13)	C10—C11—C14	110.11 (19)
C5—N5—C6	129.31 (15)	C13—C11—C14	107.4 (2)
C4—N5—C6	124.70 (15)	O6—C15—O7	109.46 (17)
C21—O10—H10	106.4 (17)	O6—C15—H15B	111.4 (14)
C2—N1—H1C	118.2 (13)	O7—C15—H15B	106.6 (14)
C2—N1—H1B	121.6 (14)	O6—C15—H15A	108.1 (13)
H1C—N1—H1B	119.5 (19)	O7—C15—H15A	103.0 (13)
C15—O6—P1	124.52 (15)	H15B—C15—H15A	117.8 (18)
C9—O3—P1	122.53 (14)	O8—C16—O7	121.8 (2)
C4—C3—N4	109.79 (13)	O8—C16—C17	125.3 (2)
C4—C3—C2	116.20 (15)	O7—C16—C17	112.77 (19)
N4—C3—C2	134.00 (15)	O5—C10—O4	121.8 (2)
C1—N2—C2	118.43 (15)	O5—C10—C11	125.9 (2)
C1—N3—C4	110.16 (15)	O4—C10—C11	112.3 (2)
N3—C4—N5	126.26 (14)	C17—C18—H18A	109.5
N3—C4—C3	127.50 (14)	C17—C18—H18B	109.5
N5—C4—C3	106.24 (14)	H18A—C18—H18B	109.5
C10—O4—C9	117.94 (19)	C17—C18—H18C	109.5
C16—O7—C15	117.27 (18)	H18A—C18—H18C	109.5
N4—C5—N5	113.69 (15)	H18B—C18—H18C	109.5
N4—C5—H5	125.8 (11)	C11—C14—H14A	109.5
N5—C5—H5	120.5 (11)	C11—C14—H14B	109.5
N1—C2—N2	118.11 (15)	H14A—C14—H14B	109.5
N1—C2—C3	123.89 (16)	C11—C14—H14C	109.5
N2—C2—C3	118.00 (14)	H14A—C14—H14C	109.5
N5—C6—C7	113.36 (15)	H14B—C14—H14C	109.5
N5—C6—H6A	106.7 (12)	C11—C12—H12A	109.5
C7—C6—H6A	108.1 (12)	C11—C12—H12B	109.5
N5—C6—H6B	104.9 (12)	H12A—C12—H12B	109.5
C7—C6—H6B	110.5 (12)	C11—C12—H12C	109.5
H6A—C6—H6B	113.5 (17)	H12A—C12—H12C	109.5
O2—C7—C6	108.72 (15)	H12B—C12—H12C	109.5
O2—C7—H7A	108.3 (11)	C19—C17—C18	114.6 (3)
C6—C7—H7A	110.1 (10)	C19—C17—C16	110.5 (3)
O2—C7—H7B	105.4 (11)	C18—C17—C16	112.3 (2)
C6—C7—H7B	107.8 (11)	C19—C17—C20	108.7 (4)
H7A—C7—H7B	116.2 (15)	C18—C17—C20	104.0 (4)
C21—C22—C22^i^	113.30 (19)	C16—C17—C20	106.1 (3)
C21—C22—H22A	106.6 (15)	C17—C20—H20A	109.5
C22^i^—C22—H22A	111.2 (15)	C17—C20—H20B	109.5
C21—C22—H22B	107.6 (12)	H20A—C20—H20B	109.5
C22^i^—C22—H22B	109.4 (12)	C17—C20—H20C	109.5
H22A—C22—H22B	108.6 (18)	H20A—C20—H20C	109.5
O9—C21—O10	122.58 (16)	H20B—C20—H20C	109.5
O9—C21—C22	123.77 (16)	C17—C19—H19A	109.5
O10—C21—C22	113.65 (15)	C17—C19—H19B	109.5
O2—C8—P1	108.92 (13)	H19A—C19—H19B	109.5
O2—C8—H8A	112.5 (14)	C17—C19—H19C	109.5
P1—C8—H8A	111.6 (15)	H19A—C19—H19C	109.5
O2—C8—H8B	111.4 (14)	H19B—C19—H19C	109.5
P1—C8—H8B	109.8 (14)	C11—C13—H13A	109.5
H8A—C8—H8B	102.5 (19)	C11—C13—H13B	109.5
N3—C1—N2	129.67 (18)	H13A—C13—H13B	109.5
N3—C1—H1A	115.2 (12)	C11—C13—H13C	109.5
N2—C1—H1A	115.1 (12)	H13A—C13—H13C	109.5
O4—C9—O3	107.72 (17)	H13B—C13—H13C	109.5
O4—C9—H9B	111.6 (16)		

Symmetry code: (i) −*x*+2, −*y*−1, −*z*.

### Hydrogen-bond geometry (Å, º)

**Table:**

*D*—H···*A*	*D*—H	H···*A*	*D*···*A*	*D*—H···*A*
N1—H1*C*···O1^ii^	0.812 (19)	2.14 (2)	2.941 (2)	170.16 (18)
N1—H1*B*···O9	0.79 (2)	2.05 (2)	2.842 (2)	175 (2)
O10—H10···N4	0.84 (2)	1.91 (2)	2.734 (2)	166 (2)

Symmetry code: (ii) *x*+1, *y*−1, *z*.
